# Relationship Between Perceived Parental Academic Expectations and Students' Self-Regulated Learning Ability: A Cross-Sectional Study

**DOI:** 10.3389/fpsyg.2022.786298

**Published:** 2022-07-14

**Authors:** Ling Xu, Lijun Ma, Peng Duan

**Affiliations:** ^1^School of Business, Liaocheng University, Liaocheng, China; ^2^School of Education, The Philippine Women's University, Manila, Philippines; ^3^School of Computer Science, Liaocheng University, Liaocheng, China

**Keywords:** perceived parental academic expectations, mastery goal orientation, self-regulated learning ability, self-reflection, college students

## Abstract

In the field of self-regulated learning ability for college students, prior research usually neglected the role of parents when considering the physical distance between college and home. Indeed, an underlying describable driving force of learning remains embedded in the relationship between parents and students, even at the college stage. This study aimed to explore the relationships among perceived parental academic expectations, mastery goal orientation, self-reflection, and self-regulated learning ability of college students. Mediation and moderated mediation analyses were employed to analyze a sample of 691 college students. The results indicate that mastery goal orientation partially mediates the relationship between perceived parental academic expectations and self-regulated learning. In addition, self-reflection moderates the relationship between perceived parental academic expectations and self-regulated learning. The positive correlation between perceived parental academic expectation and self-regulated learning is stronger for students with low self-reflection than those with high self-reflection.

## 1. Introduction

Self-regulated learning (SRL) is an active, motivational and volitional learning process that includes but is not limited to metacognitive strategies, goal setting, time management, task strategies, environment structuring, and self-reflection (Schunk and Zimmerman, 2007). As an important learning ability, especially for college students, SRL not only helps them to study well in school but also has a profound impact on their lifelong development after leaving school. Researchers have proposed many models to illustrate the working mechanism of SRL. For example, Winne and Hadwin (2013) determined that the SRL process has five parts: task interpretation, learning goal setting, learning strategy selection, strategy application, and learning result obtainment. Pintrich (2000) presented a learning conceptual framework model that divided SRL into four stages: thinking activation, monitoring, control, and reflection. Rozendaal et al. (2005) investigated a dual SRL model that described the interaction between the growth goals and the wellbeing goals. Zimmerman (2000) developed a social cognitive model that divided SRL into three stages: planning, behavior or volition control stage, and self-reflection. From the perspective of the aforementioned SRL models, although models have some differences in the details, most emphasize the importance of initiative, goal orientation, and self-control in the learning process. Meanwhile, another common feature of these models is that they ignore the importance of the social environment (peers, teachers, parents, etc.) in self-regulation and its development (Panadero and Alonso Tapia, 2014). Aware of this limitation, numerous researchers focused their attention on the learner's surrounding environment, such as classmates, friends, teachers, and family members (Panadero et al., 2016). There is strong evidence that cultural values play an important role in educational achievements. For example, Asian students are always taught to obey their parents, respect their elders, and establish a good family reputation by making outstanding achievements in life (Zhang and Carrasquillo, 1995). Indeed, compared with teachers and peers, parents have a lasting and profound influence on their children. For most students, as they grow older, their sense of responsibility to build a good family reputation significantly increases, which will motivate them to work hard to meet their parents' requirements or expectations (LaRocque et al., 2011). This is a striking indication of how parents play a significant role in their children's educational achievement. Increasing numbers of researchers have focused on the influence of parents' management of their children's education, parents' expectations, and parents' participation in school activities on the academic performance of primary and secondary school students (Jaiswal and Choudhuri, 2017). However, current research lacks exploration and further explanation of the relationship between parental academic expectations and students' SRL ability, especially for college students.

Mastery goal orientation focuses on developing of competence and task mastery and leads to positive educational outcomes, including human cognition, behavior, persistence, and emotions (Nelson et al., 2015). Most researchers believed that goal orientation was characteristic, stable, and derived from individual differences, yet it was difficult to explain the underlying process and dynamics. Button et al. (1996) elaborated that goal orientation as a state variable instead of a constant structure and proposed that individuals could selectively employ goal orientation. Cerasoli and Ford (2014) found that students who adopted a mastery goal orientation had a generally positive patterns of motivational beliefs, including adaptive levels of task value, self-efficacy, and academic performance. In addition, Kaplan and Flum (2010) confirmed the potential reciprocal relationship between adolescents' academic achievement goal orientation and their identity formation style. However, recent studies mainly emphasized the important role of goal orientation in the growth of the individual, without elaborating the formation mechanism of goal orientation. Hence, the relevant factors of individual goal orientation have become popular in the research field.

Since the expectation effect theory was proposed by Rosenthal and Jacobson (1968), the role of expectations has received increasing attention from researchers. Relevant work includes teachers' expectations for students and parents' expectations for children (Zhang, 2015). For example, Benner and Mistry (2007) found that teachers' expectations were related to students' perceptions of their math reading skills and ability to learn new concepts. Similarly, parents' academic expectations have been a variable in many studies of parental involvement. Compared with other types of parental involvement, such as participation in school activities, parent-child communication, and homework help, parents' academic expectations had the greatest impact on academic performance (Jaiswal and Choudhuri, 2017). Studies find that students whose parents have high expectations receive better grades than those whose parents have low expectations (Yamamoto and Holloway, 2010). As we know, due to the distance from their family and the associated daily constraints, college students exhibit diverse learning styles and are independently in control of their learning time; as such, students' academic performance is more closely related to their SRL ability (Wolters and Hussain, 2015). However, regarding SRL ability, few studies investigated the effect of parents' expectations on young adults, especially college students. Compared with primary and secondary school students, college students are more likely to self-reflect when their parents' expectations are inconsistent with their academic performance (Wang and Heppner, 2002). Self-reflection in SRL refers to the processes of learner looking back on their past learning experiences, including what they did to enable learning to occur, and exploring connections between the knowledge that was taught and the learner's ideas (Lew and Schmidt, 2011). Zimmerman and Schunk (2011a) found that self-reflection was an important factor in SRL that regulates learners' behaviors.

In this paper, we first investigate the association between students' perceived parental academic expectations and students' SRL ability. Then, we analyze the mediating role of mastery goal orientation between perceived parental academic expectations and students' SRL ability. Third, we explore the moderating role of self-reflection in the direct correlation between perceived parental academic expectations and students' SRL ability.

### 1.1. Perceived Parental Academic Expectations and Students' SRL Ability

Parents' academic expectations drive children's behavior and promote their success in school and life (Wang, 2015). According to the expectation effect theory, human expectations, as a changeable psychological state, not only promotes but also influences others' behaviors (Rosenthal and Jacobson, 1968). The ecological systems theory emphasizes that personal growth is nested in series of environmental systems influencing each other, and under that theory, parents play a significant role in the growth of their children (Guy-Evans, 2020). Many subsequent studies support that parents' educational expectations influence their children's academic performance (Cheng and Starks, 2002; Yamamoto and Holloway, 2010). In general, students' academic performance is inseparable from their SRL ability, especially for college students.

### 1.2. Perceived Parental Academic Expectations, Students' SRL Ability, and Mastery Goal Orientation

Goal orientation guides human thought, action, and emotion, as well as individual behaviors (Kaplan and Maehr, 2007). Students dominated by mastery goal orientation are likely to learn new skills, master new methods, and improve their abilities (Dweck et al., 2004). Almost all researchers believe that mastery goal orientation has positive effects on individual cognition, motivation, self-reflection, deep learning, perseverance, and performance (Cerasoli and Ford, 2014). As a personality trait, individual mastery goal orientation has both a stable and an unstable side. Elliot and Thrash (2001) pointed out that individual goal was influenced by the environment. Researchers also found that students' mastery goal orientation enhanced their self-regulation (Zimmerman and Schunk, 2011b). Different environmental conditions affect individuals' different goals and behaviors (Murayama and Elliot, 2009). Parents, teachers, and friends are environmental factors that influence students' SRL ability, among which the educational perspective of parents plays a leading role (Daniel et al., 2016). Influenced by traditional culture, Chinese students' goal orientation often directly or indirectly reflects the wishes of their parents. Students usually respect the opinions of their parents and consult their parents' ideas when setting learning goals. Parents express expectations for their children without reservation in all areas. As a sensitive motivating factor of SRL ability, such expectations from parents have a potential promoting effect on students' mastery goal orientation that could mediate the relationship between perceived parental academic expectations and students' SRL ability. Considering the above arguments, we proposed the following hypothesis:

**Hypothesis 1**: Mastery goal orientation mediates the relationship between perceived parental academic expectations and students' SRL ability.

### 1.3. The Moderating Role of Students' Self-Reflection

Self-reflection is the inspection and evaluation of individual thought, feeling, behavior, and insight. It is a metacognition of change processes with purpose and direction (Scheier and Carver, 1983). Relevant research on self-reflection mainly focuses on positive psychological qualities, such as self-development, self-integrity, and cognition (Fernández-Álvarez et al., 2015; Abitova et al., 2020). However, researchers have also found a significant positive correlation between individual self-reflection and problem coping strategies (Burwell and Shirk, 2007). Meanwhile, students with high self-reflection are good at evaluating their thoughts, feelings, and behaviors, which enables them to make strategic adjustments to situational changes (Stefan and Cheie, 2022). Indeed, individuals' self-reflection is correlated with their metacognition, learning strategies, and future achievement. In the systemic self-reflection model, self-reflection is more likely to occur during moderate stress to regulate individual thoughts and behavior; thus, adaptive self-reflection is more likely to occur in response to daily stressful events (Crane et al., 2019). Students who are learning under strong parental academic expectations are used to comparing their academic performance with parents' expectations and reflecting on their learning behavior, which could help further improve their SRL ability. Considering the above arguments, we proposed the following hypothesis.

**Figure 1:**
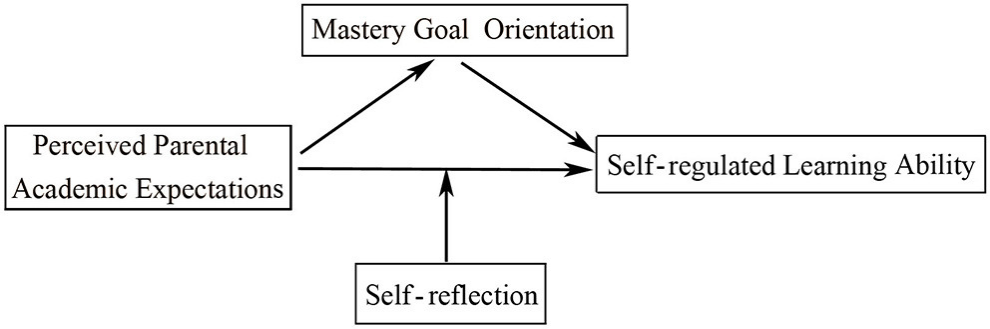
The hypothesis model of the relationships between perceived parental academic expectations, mastery goal orientation, self-regulated learning ability, and self-reflection.

**Hypothesis 2**: Students' self-reflection moderates the strength of the direct association between perceived parental academic expectations and students' SRL ability. Figure 1 illustrates the proposed model.

## 2. Methods

### 2.1. Study Design

This study was based on cross-sectional data collected from 691 college students in China. All participants came from cities in Shandong Province.

### 2.2. Participants and Data Collection

We distributed 720 questionnaires, and 691 usable questionnaires were returned (the efficiency response rate was 96%). Among the students, 59% (*n* = 408) were female, and 41% (*n* = 283) were male. As for their age, 100% (*n* = 691) of the students were aged 17–24, and their average age was 19.49 years old (*SD* = 0.526). In the survey, the students completed the questionnaires to measure their mastery goal orientation levels, the actual states of their SRL ability, their self-reflection levels, and the perceptions of their parents' expectations. We explained the survey purpose to all the students before they completed the questionnaire. We obtained approval from the ethics committee of Liaocheng University. The participants filled out the surveys anonymously and voluntarily with no negative consequences for not filling.

### 2.3. Instruments

The questionnaire items were written in English and translated into Chinese. To ensure cross-linguistic equivalence, the results were independently translated back to English by two English professors.

#### 2.3.1. Perceived Parental Academic Expectations

We measured the students' perceived parental academic expectations using the modified Parental Expectations Scale (PES) (Eisen et al., 2004). The PES is a 20-item parents' self-report instrument with items rated on 5-point Likert-type scales ranging from 1 (not very true) to 5 (extremely true). The PES assesses parents' expectancies in five dimensions, namely, academic achievement, extracurricular activities, household responsibilities, peer activities, and general success. Because we were only concerned with the academic expectations, we only administered the portion of the PES that addressed academic achievements (e.g., “My parents think academic success is an important goal for me.”). For this study, the students completed the scale themselves rather than their parents. We employed confirmatory factor analysis (CFA) to test all variables' construct validity and used the standards for good fit to determine whether the variable had good structural validity (Hu and Bentler, 1998): *X*^2^/*df* < 5, root mean square error of approximation (RMSEA) < 0.1, and comparative fit index (CFI) > 0.90. The perceived parental academic expectations indicators of CFA were *X*^2^/*df* = 3.98, RMSEA = 0.07, and CFI = 0.99. The Cronbach's α for the measure of perceived parental academic expectations is 0.84.

#### 2.3.2. Mastery Goal Orientation

To assess students' mastery goal orientation, we administered the Intrinsic Goal Orientation Scale (IGOS) in the Motivated Strategies for Learning Questionnaire Manual (Pintrich et al., 1991). This scale includes four items that concern students' reasons for their task participation. An example item from the IGOS is “I prefer subjects with challenging contents, so I can learn new knowledge.” The responses are rated on a 5-point Likert scale ranging from 1 (none at all) to 5 (a great deal). CFA results showed that the fit indices were *X*^2^/*df* = 1.92, RMSEA = 0.04, and CFI = 0.99. The Cronbach's α for mastery goal orientation is 0.79.

#### 2.3.3. Self-Regulated Learning Ability

The SRL ability scale was taken from Zhang and Li (2007). This scale has 30 items measuring four dimensions: learning motivation (8 items), self-management ability (11 items), cooperative learning ability (5 items), and information quality (6 items). An item from this scale is “I pay more attention to mastering professional knowledge and skills instead of passing examinations.” Items are rated on a 5-point Likert scale. The SRL variables exhibited good convergent validity indicated by the fit indices: *X*^2^/*df* = 2.9, RMSEA = 0.05, and CFI = 0.94. The Cronbach's α for SRL ability is 0.78, and the Cronbach's α values for the dimensions of SRL ability ranged from 0.74 to 0.82. Hence, this was an appropriate survey for measuring SRL ability.

#### 2.3.4. Self-Reflection

We used two of the subscales of the Self-reflection and Insight Scale (Grant et al., 2002) to measure the students' self-reflection. The subscale items are divided into two dimensions: active reflection and analytic reflection. Active reflection is the self-exploration of human thought, feeling, and behaviors, for instance, “I frequently take time to reflect on my thoughts.” Analytic reflection means the deep contemplation of individual thought, feeling, and actions, for instance, “It is important for me to evaluate the things that I do.” We used the average score of all items to indicate the students' self-reflection. The indicators of validity using CFA were *X*^2^/*df* = 3.0, RMSEA = 0.05, and CFI = 0.97. The Cronbach's α for self-reflection is 0.91, and the Cronbach's α values for the dimensions of self-reflection ranged from 0.88 to 0.92.

#### 2.3.5. Controls

We measured and controlled for differences of participants, including age, gender in the mediation and moderated mediation analyses in this study.

### 2.4. Data Analysis and Availability

We used SPSS 22.0 for partial correlation analysis (age is controlled) to determine the relationships among perceived parental academic expectations, students' SRL ability, mastery goal orientation, and self-reflection in females and males, respectively. We then used mediation modeling analysis (Model 4, SPSS 22.0 PROCESS macro) to test whether mastery goal orientation mediated the association between perceived parental academic expectations and SRL ability. Finally, we employed moderated mediation modeling analysis (Model 59) to test for a moderating effect of students' self-reflection on the proposed mediation model. All variables were centralized to reduce collinearity in the moderation analysis. The bootstrap confidence interval (CI) for the indirect effect was estimated based on 5,000 bootstrap samples and was bias-corrected. In all statistical analyses, *p* < 0.05 was significant.

## 3. Results

### 3.1. Descriptive and Correlation Statistics

Table 1 summarizes the means, standard deviations, and correlations among the study variables. From Table 1, we can observe that perceived parental academic expectations are positively related to students' mastery goal orientation and SRL ability. The results also indicate that self-reflection is positively correlated with parents' academic expectations and students' mastery goal orientation. In addition, we can see that the correlations among these variables are different in female and male students. In detail, female students' perceived academic expectations are more related with mastery goal orientation, SRL ability, and self-reflection. This could be because female students are emotionally closer to their parents than male students in daily life (Sax and Weintraub, 2014). However, male students' mastery goal orientation was related more with SRL ability and self-reflection. In addition, the correlation between self-reflection and SRL ability in male students was more significant than that in female students.

**Table 1:** Means, standard deviations, and correlations of all measures.

	**Gender**	**Mean**	**SD**	**1**	**2**	**3**
1. Perceived parental academic expections	Females	4.09	0.44			
	Males	4.06	0.45			
2. Mastery goal orientation	Females	3.87	0.58	0.453^**^		
	Males	3.97	0.66	0.448^**^		
3. SRL ability	Females	3.98	0.49	0.556^**^	0.711^**^	
	Males	3.96	0.63	0.539^**^	0.733^**^	
4. Self-reflection	Females	3.70	0.40	0.558^**^	0.519^**^	0.642^**^
	Males	3.58	0.44	0.520^**^	0.577^**^	0.691^**^

*n = 691;*

** *p < 0.01*.

### 3.2. Mediation Model Analysis

Hypothesis 1 proposed that mastery goal orientation would mediate the link between perceived parental academic expectations and students' SRL ability. To examine this hypothesis, we employed Model 4 in the SPSS macro to examine the mediation effect under the control of gender and age, and the mediation model was significant: *R*^2^ = 0.58, *F* = 240.94, *p* < 0.001. Table 2 demonstrates that students' perceived parental academic expectations are significantly associated with their SRL ability (*b* = 0.37, *p* < 0.001; see Model 2 of Table 2). Students' perceived parental academic expectations was significantly associated with their mastery goal orientation (*b* = 0.65, *p* < 0.001; see Model 1 of Table 2). Students' mastery goal orientation was significantly associated with their SRL ability (*b* = 0.53, *p* < 0.001; see Model 2 of Table 2).

**Table 2:** The mediation effect of mastery goal orientation.

**Predictors**	**Model 1 (MGO)**		**Model 2 (SRL)**	
	**b**	**t**	**b**	**t**
PPAE	0.65	13.30^***^	0.37	10.15^***^
MGO			0.53	21.07^***^
*R* ^2^	0.23		0.58	
*F*	67.57^***^		240.94^***^	

*n = 691; Each column is a regression model that predicts the criterion at the top of the column. PPAE, perceived parental academic expectation; MGO, mastery goal orientation; SRL, self-regulated learning*.

*** *;p < 0.001*.

The bootstrapping results in Table 3 indicate that the direct effect (0.37) and intermediate effect (0.34) accounted for 52.11 and 47.89% of the total effect (0.71), respectively. In addition, the upper and lower bounds of the 95% CI for the indirect effect of students' perceived parental academic expectations on their SRL ability ranged from 0.29 to 0.40, not containing zero (Table 3). In short, these findings reflect an association of perceived parental academic expectations and students' SRL ability. Furthermore, mastery goal orientation plays a mediating role in regulating this association. Therefore, hypothesis 1 of a mediating effect is supported.

**Table 3:** Distribution of the total effect, direct effect, and intermediary effect.

	**Effect**	**SE**	**LLCI**	**ULCI**	**Effect proportion (%)**
Total effect	0.71	0.04	0.63	0.79	
Indirect effect	0.34	0.03	0.29	0.40	47.89
Direct effect	0.37	0.04	0.30	0.44	52.11

*n = 691; CI, confidence interval; LL, lower limit; UL, upper limit*.

### 3.3. Testing for Moderated Mediation Effect

**Figure 2:**
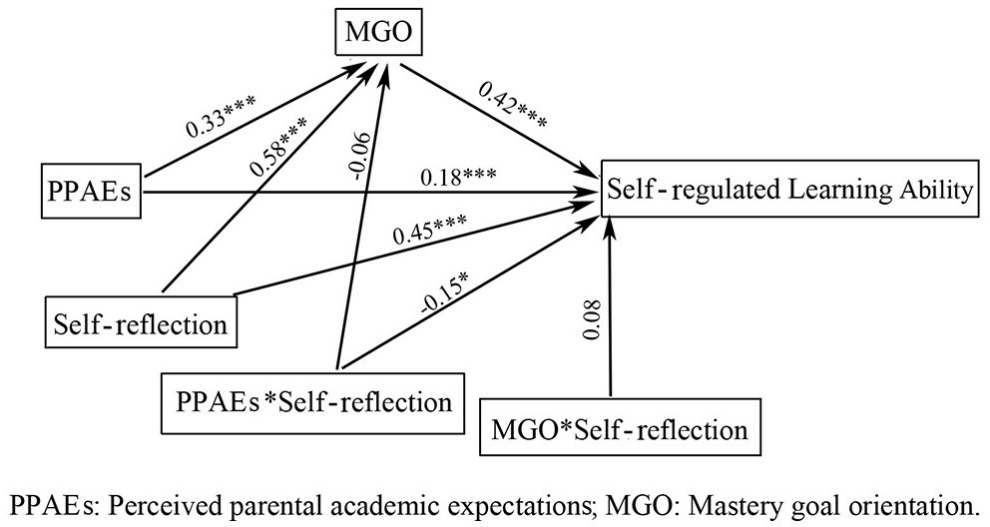
Statistical diagram of moderated mediation model in this study. ^*^p < 0.05, ^***^p < 0.001.

To examine the moderating role of self-reflection, we employed the PROCESS macro Model 59 developed by Hayes and Preacher (2013). Figure 2 and Table 4 present the path results of the hypothesized moderated mediation model, and the final model was significant: *R*^2^ = 0.65, *F* = 181.52, *p* < 0.001. Figure 2 illustrates all statistically significant paths with standardized beta (*b*) weights in the final model. Self-reflection significantly moderated the association between perceived parental academic expectations and SRL ability (*b* = −0.15, *p* < 0.05). However, the moderating effect of self-reflection on the link between perceived parental academic expectations and mastery goal orientation on SRL ability was not significant.

**Table 4:** Testing the moderated mediation effect of perceived parental academic expectation on SRL ability.

**Predictors**	**Model 1 (MGO)**		**Model 2 (SRL)**	
	**b**	**t**	**b**	**t**
PPAE	0.33	5.93^***^	0.18	4.85^***^
SR	0.58	10.50^***^	0.45	11.34^***^
PPAE × SR	−0.06	−0.62	−0.15	−2.03^*^
MGO			0.42	16.95^***^
MGO × SR			0.08	1.52
*R* ^2^	0.34		0.65	
*F*	69.62^***^		181.52^***^	

*n = 691. PPAE, perceived parental academic expectation; MGO, mastery goal orientation; SRL, self-regulated learning*;

* *p < 0.05*,

*** *p < 0.001*.

**Figure 3:**
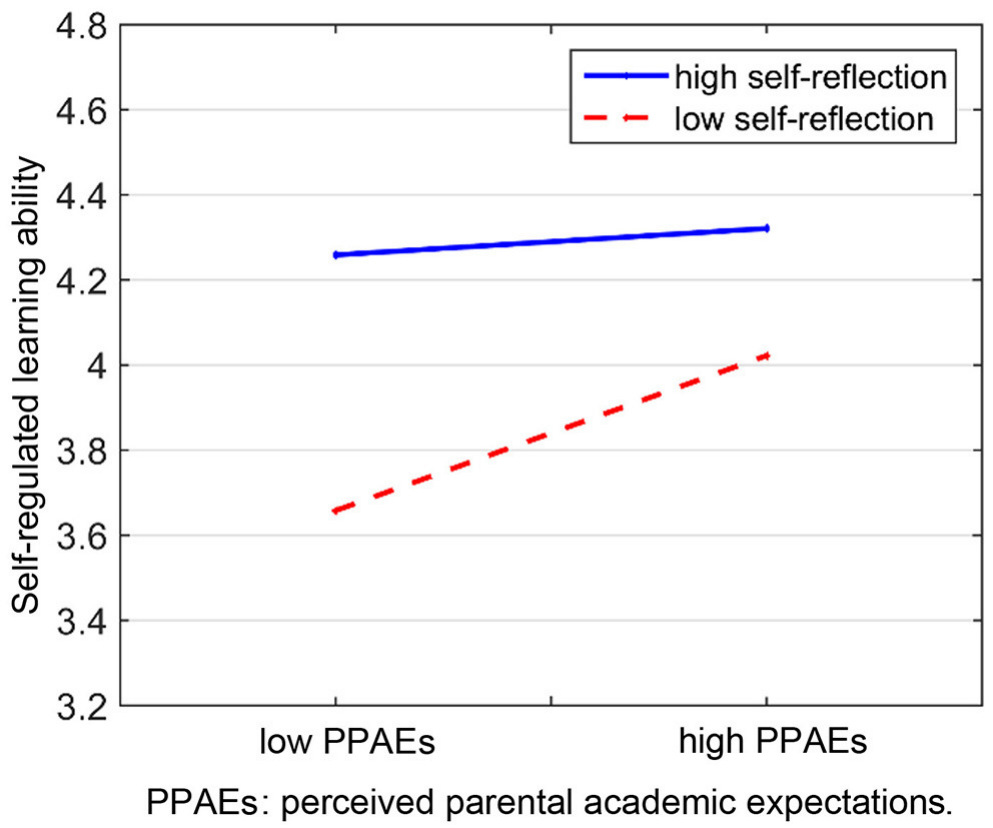
Simple slopes of perceived parental academic expectations (PPAEs) predicting SRL ability with low (one SD below M) and high (one SD above M) levels of self-reflection.

The interaction effect demonstrated that as the self-reflection levels increased, the effect of perceived parental academic expectations on SRL ability decreased. Table 5 shows that the conditional direct effect of perceived parental academic expectations on students' SRL ability was much stronger among students with low self-reflection (conditional direct effect = 0.25 and *p* < 0.01, with 95% CIs of 0.16–0.33), compared with students who showed moderate (conditional direct effect = 0.18 and *p* < 0.01, with 95% CIs of 0.11–0.26) and high (conditional direct effect = 0.12 and *p* < 0.05, with 95% CIs of 0.01–0.23) self-reflection. Figure 3 presents the interaction pattern of perceived parental academic expectations with students' SRL ability at different self-reflection levels. The results indicate that the students with high level self-reflection have significantly stronger SRL ability than those with low level self-reflection. In addition, as perceived parental academic expectations increased, the students' SRL ability scores increased more for the students with low level self-reflection than those with high level self-reflection. This finding suggests that perceived parental academic expectations relate to student's SRL ability more significantly among students with low level self-reflection. In sum, self-reflection acted as a moderator of the relationship between perceived parental academic expectations and students' SRL ability.

**Table 5:** Results of the conditional direct effect of perceived parental academic expectation on students' SRL ability across different levels of self-reflection.

**Self-reflection**	**Mean**	**Conditional**	**t**	**p**	**LLCI**	**ULCI**
**level**		**direct effect**				
Low (M-1SD)	−0.42	0.25	5.80^***^	≤ 0.01^**^	0.16	0.33
Moderate level	0	0.18	4.85^***^	≤ 0.01^**^	0.11	0.26
High (M+1SD)	0.42	0.12	2.16^*^	0.03^*^	0.01	0.23

*n = 691*;

* *p < 0.05;*

** *p < 0.01*;

*** *p < 0.001*;

*CI, confidence interval; LL, lower limit; UL, upper limit*.

## 4. Discussion

Previous studies have demonstrated that parents' academic expectations influence students' academic achievements, especially for primary and secondary school students (Nihal Lindberg et al., 2019). However, for college students, most studies emphasized the psychological pressure and anxiety caused by parents' academic expectations while ignoring the positive role (Cooper and Cefai, 2013). In view of the significant positive correlation between academic performance and SRL ability (Hong and Park, 2012), this study aimed to investigate the effect of perceived parental academic expectations on students' SRL ability through mastery goal orientation. Our findings show a positive correlation between perceived parental academic expectations and students' SRL ability, indicating the importance of parents' participation in cultivating students' SRL ability. The findings that students working under their parents' high level academic expectations demonstrate high SRL ability is consistent with previous findings that parents' academic expectations have long-lasting influence on children's future achievements (Yamamoto and Holloway, 2010).

Scholars believe that the positive effect of parents' academic expectations on students' SRL ability could be related to students' positive self-motivation (Harackiewicz et al., 2000; Linnenbrink-Garcia et al., 2008). As parents' academic expectations are a continuous and stable incentive, they can easily have profound effects on students' academic career. Considering the independent learning style of college students, we tend to believe that parents' academic expectations with rich emotions and family responsibilities can promote students' development toward achieving their parents' desired goals for them. Indeed, the greater parents' emotional investment (or emotional labor) in their children's education, such as maintaining a harmonious family atmosphere, the more likely their children will succeed in education as a kind of emotional support (Wong, 2022). As we know, goal orientation is an intrinsic belief easily influenced by environmental factors e.g., parents' perspectives and teachers' policies and procedures (Geitz et al., 2016). For example, Panadero (2017) demonstrated that SRL ability was a goal-driven process and correlated with motivation. Maehr and Zusho (2009) found that mastery goal orientation was associated with high academic achievements. In this study, we confirmed multiple associations among parents' academic expectations, students' mastery goal orientation, and SRL ability. In detail, perceived parental academic expectations have a positive relationship with students' SRL ability *via* mastery goal orientation.

Annual reports from many universities have indicated that external expectations such as parental attitude and high parental expectations, cause stress among school students; academic expectations that are too heavy to have a negative influence, leading to severe anxiety and sense of failure (Deb et al., 2015). We found that self-reflection helps students confidently adapt to their parents' academic expectations. Students with high self-reflection level seem to have lower acceptance of parental expectations than those with low self-reflection (Frederick, 2005). One interesting finding was that the direct correlation between parents' academic expectations and SRL ability was stronger for students with low self-reflection level than those with high self-reflection level. High self-reflection might reduce excessive pressure caused by high academic expectations.

The contributions of this paper are as follows: (1) This study enriches the discussion of social environmental factors in the theoretical model of self-regulated learning by identifying that perceived parental academic expectations are positively associated with students' SRL ability. (2) We explained a new indirect association between perceived parental academic expectations and students' SRL ability *via* mastery goal orientation. Furthermore, we examined the moderating effect of self-reflection in the direct association between perceived parental academic expectations and students' SRL ability. (3) These findings have implications for parents' involvement in their children's education. Studies have demonstrated that an autonomy-supportive parenting style effectively improved parents' remote supervision, and established the compatibility of supervision and autonomy support (Rodŕıguez-Meirinhos et al., 2020). Parents could actively experiment with the autonomy-supportive parenting style. Autonomy support acts on children's proximal development zone, which enhanced their motivation and self-efficacy and helped them better achieve parents' expectations (Distefano et al., 2018; Moè et al., 2018). With parents' autonomy support, children feel their parents' care and listening, which alleviates pressure and anxiety caused by their studies (Moe et al., 2020). Based on this research, parents can provide an atmosphere of emotional warmth and support in which high academic expectations are conveyed, students should show higher motivation under greater academic expectations from their parents. It should be noted that studies have demonstrated that parents' high level academic expectations can be detrimental to students' mental health. Thus, parents need to know their children's abilities and maintain their academic expectations within a certain range to avoid inducing academic stress and anxiety. Parents also need to pay attention to cultivating children's self-reflection ability. In goal guidance, parents and teachers focus on cultivating children for the purpose of mastering knowledge. In this way, children often hold positive, adaptive patterns of intrinsic motivation. The intrinsic motivation model enables students to effectively use deep processing strategies, while maintaining positive emotions, generating lasting motivation, and promoting students to achieve greater achievements.

## 5. Limitations and Future Research Suggestions

One shortcoming of this research is that we studied the relationship between perceived parental academic expectations and students' SRL ability but only focused on college students; we did not include primary and middle school students in the research scope. Compared with primary and middle school students, college students have stronger self-reflection ability and higher independent SRL ability; thus, the effect of perceived parental academic expectations on college students is different from that on younger students. For this reason, these research results might not be generalizable. Another shortcoming of this research was that this is cross-sectional rather than longitudinal. The study results show relationships among the variables, but we did not verify causality. The findings from the cross-sectional model presented in this paper indicate that parental academic expectations, as an environmental factor, influence children's development. Parental academic expectations cultivated students' SRL ability by strengthening their mastery goal orientation. Previous research on goal orientation has demonstrated that individual goals are susceptible to external and internal factors (Manganelli et al., 2015; Hadwin et al., 2017), and there are strong correlations between motivational variables and deep learning strategies (Cassidy, 2011; Diseth, 2011). For example, in a mastery goal-oriented classroom, students' personal goals tend to converge in the same direction. This phenomenon demonstrates that motivation has a positive impact on students' academic performance (Alivernini et al., 2018). In addition, research found that individual motivation presented different responses in different student groups (Manganelli et al., 2021). Specifically, compared with peers with high socioeconomic status, adolescents with low socioeconomic status had lower intrinsic motivation and recognition regulation (Alivernini et al., 2019). In addition, personality traits, self-esteem, critical thinking, and self-efficacy belief also have an impact on motivation (Alivernini et al., 2021). Moreover, autonomic forms of support from parents and teachers promoted autonomic motivation and self-efficacy, which in turn predicted the academic adjustment of first-year university students (Girelli et al., 2018). Educators should pay more attention to students' personality antecedents, and then adjust their motivation and learning activities (Di Giunta et al., 2013; Manganelli et al., 2019; Samuel and Burger, 2020). Hence, in future studies, we will try our best to analyze the potential effect of confounding factors, including achievement goals and motivation, personality, teacher support, and other variables, on students' SRL ability. Third, we collected all questionnaire data as self-reports by the students themselves, including on their parents' academic expectations. Future research could also seek to identify differences between parents' own self-reported academic expectations and what their children perceive through interviews or parents' reporting to achieve more effective transmission of academic expectations between parents and children.

## 6. Conclusion

In this study, when students perceived high parental academic expectations, their SRL ability increased through mastery goal orientation, especially for those with low self-reflection level. Perceived parental academic expectations are directly and positively correlated with students' SRL ability, and mastery goal orientation partly mediates that relationship. Self-reflection significantly moderated the association between perceived parental academic expectations and students' SRL ability. In conclusion, parental academic expectations can be useful for building in students a mastery goal orientation and improving their SRL ability.

## Data Availability Statement

The raw data supporting the conclusions of this article will be made available by the authors, without undue reservation.

## Author Contributions

LX provided substantial contributions to the research conception and design. LX and LM analyzed and interpreted the data. LX and PD wrote the paper. All authors contributed to the article and approved the submitted version.

## Funding

This research was partially supported by Teaching Reform Project of Liaocheng University (G202119, G202120, G202103, and G201721), Natural Science Foundation of Shandong Province (ZR2016FL13), Doctoral Research Foundation Project of Liaocheng University (318051532), and the Key Teaching Reform Project of Shandong Province (Z2021159).

## Conflict of Interest

The authors declare that the research was conducted in the absence of any commercial or financial relationships that could be construed as a potential conflict of interest.

## Publisher's Note

All claims expressed in this article are solely those of the authors and do not necessarily represent those of their affiliated organizations, or those of the publisher, the editors and the reviewers. Any product that may be evaluated in this article, or claim that may be made by its manufacturer, is not guaranteed or endorsed by the publisher.
